# Isolation, Characterization, and Antioxidant Activity of Peptides Derived from *Bangia fuscopurpurea*

**DOI:** 10.3390/foods14244220

**Published:** 2025-12-09

**Authors:** Jiaying Ke, Nan Jia, Yusong Qiu, Chao Zhao

**Affiliations:** 1 Biomedical Department, Huaqiao University, Quanzhou 362021, China; kejiaying2003@163.com; 2 College of Oceanology and Food Science, Quanzhou Normal University, Quanzhou 362000, China; 3 College of Marine Sciences, Fujian Agriculture and Forestry University, No. 15 Shangxiadian Road, Fuzhou 350002, China; jn17703482020@163.com; 4 College of Food Science, Fujian Agriculture and Forestry University, Fuzhou 350002, China; yusongqiu316@163.com

**Keywords:** *Bangia fuscopurpurea*, antioxidant peptide, oxidative stress, HepG2, *Caenorhabditis elegans*

## Abstract

Oxidative stress is related to cellular damages and aging. Bioactive peptides have the potential to be a useful functional ingredient, although *Bangia fuscopurpurea* (red alga), a dense protein source, has not yet been exploited as a source of antioxidant peptide. The aim of the study was to prepare, isolate, and characterize antioxidant peptide of *B. fuscopurpurea* and assess their protection against oxidative stress in vitro, in HepG2 cells, and in *Caenorhabditis elegans*. Protein of *B. fuscopurpurea* was subjected to hydrolysis with papain and purification of the hydrolysate was performed through multi-step chromatography and ultrafiltration. The LC-MS/MS identified peptides, which were synthesized and screened. Two new peptides, YPCW and GYPYK, were discovered and both of them had strong antioxidant properties in vitro, with the ABTS radical scavenging IC_50_ of 2.52 ± 0.37 µg/mL and ORAC of 5187 ± 78 µmol TE/g. Both peptides in H_2_O_2_-induced HepG2 cells significantly decreased the intracellular ROS and MDA and inhibited the activity of antioxidant enzymes (SOD, CAT, and GSH-Px). Moreover, YPCW and GYPYK increased the survival of *C. elegans* during oxidative stress and the similar response, altering antioxidant enzyme activities in vivo and MDA levels. These findings indicate that peptides obtained through *B. fuscopurpurea* can be useful as antioxidant agents, and they can be considered as a possible new active ingredient of functional foods or pharmaceuticals to counteract oxidative stress.

## 1. Introduction

*Bangia fuscopurpurea* is a type of alga that is also referred to as *Red Hair Alga*, *Cattle Hair Alga*, or *Red Thread Alga* and is found along the southeast coast of China. It is a local distinct aquatic product which is a phylum Rhodophyta, family Bangiaceae, and genus *Bangia* [1]. *B. fuscopurpurea* is rich in nutrients and has great culinary and medical importance [2]. Studies of enzymatic hydrolysis in the production of bioactive peptides of red algae are comparatively few. Also, the remarkably high protein percentage of red algae implies wide opportunities of enzyme hydrolysis in the extraction of bioactive peptides.

Oxidative stress is caused by the disproportion between reactive oxygen species (ROS) and reactive nitrogen species generation and antioxidant defensive system [3,4]. In the case of an oxidative stress on the body, it results into oxidative damage of intracellular macromolecules, causing cellular functional impairment, aging, apoptosis, and irreversible damage to the body [5,6,7]. The effect of oxidative stress on human health cannot be ignored either in terms of nutrition or clinical aspects. The existing studies on this topic have attracted significant interest among scientists across the world.

The features of functional foods which are mainly made out of peptide compounds are one of the present burning research areas. Peptides are molecules that comprise one or more amino acids that are attached by peptide bonds in protein molecules [8,9]. Bioactive peptides, such as antioxidant peptides, anti-photoaging peptides, anti-obesity peptides, and hypoglycemic peptides, are peptides with the ability to regulate physiological processes in the body [10,11,12]. Bioactive peptides are safer, reliable, and more diverse than the amino acids or proteins because they are found to be more biologically easily absorbed. They are able to affect different organs of the human body, increase immunity, and boost the well-being of the digestive, endocrine, and nervous systems [13,14]. Currently, bioactive peptides have extensive applications in both the food and pharmaceutical industries.

This paper examined the antioxidant ability of red alga antioxidant bioactive peptides on the oxidative stress caused by hydrogen peroxide on human hepatocellular carcinoma (HepG2) cells. The study involved the preparation, isolation, and purification of these peptides, in vitro, cellular studies and studies on the nematodes. The results give new a precursor material to alleviate oxidative stress damage, and set a theoretical basis for the high-value use of red algae in pharmaceutical, cosmetic, and health foods.

## 2. Materials and Methods

### 2.1. Materials

*B. fuscopurpurea* was procured from Nanri Town (Putian, China), while HepG2 cells from the Chinese Academy of Sciences Cell Bank, and wild-type N2 *Caenorhabditis elegans* was procured from Fujian Shangyuan Biological Science & Technology Co., Ltd. (Fuzhou, China). Bromelain (CAS 37189-34-7), alkaline protease (CAS 9014-01-1), neutral protease (CAS 9068-59-1), and papain (CAS 9001-73-4) were purchased from Beijing Solarbio Science & Technology Co., Ltd. (Beijing, China); the catalase (CAT) activity assay kit, superoxide dismutase (SOD) activity assay kit, and glutathione peroxidase (GSH-Px) activity assay kit were purchased from Nanjing Jiancheng Bioengineering Institute (Nanjing, China); and DPPH (CAS No. 1898-66-4) (2,2-diphenyl-1-picrylhydrazyl), ABTS (CAS 30931-67-0) (2,2′-azino-bis(3-ethylbenzothiazoline-6-sulfonic acid) diammonium salt), AAPH (CAS No. 28752-68-3) (2,2′-azobis(2-amidinopropane) dihydrochloride), water-soluble vitamin E (CAS No. 53188-07-1) (6-hydroxy-2,5,7,8-tetramethylchroman-2-carboxylic acid), and sodium fluorescein (CAS 2321-07-5) (3′,6′-dihydroxyspiro[isobenzofuran-1(3H),9′-xanthen]-3-one) were purchased from Sigma-Aldrich China (Wuxi, China). The DMEM medium was obtained from Thermo Fisher Scientific (Beijing) Co., Ltd. (Beijing, China).

### 2.2. Equipment

The equipment used were a microplate reader (CLARIOstar, BMG Labtech, Ortenberg, Germany); freeze dryer (FDS-2110, Tokyo Rikakikai Co., Ltd., Tokyo, Japan); a Tangential Flow Ultrafiltration System (Sartorius, Göttingen, Germany) with Ultrafiltration Membrane Cassettes (Catalog Nos. 3081441902E-SW and 3081443902E-SW); a Capillary High-Performance Liquid Chromatography System (Ultimate 3000, Thermo Fisher Scientific, Waltham, MA, USA); and an Electrospray Ionization Hybrid Quadrupole–Orbitrap Mass Spectrometer (Q Exactive™ Hybrid Quadrupole-Orbitrap™ Mass Spectrometer, Thermo Fisher Scientific, USA).

### 2.3. Sample Preparation

*B. fuscopurpurea* was washed to remove impurities and then dried at 60 °C for 12 h. The dried algae were ground through an 80-mesh sieve to obtain *B. fuscopurpurea* powder, and the powder was bagged for later use.

### 2.4. Protein Extraction Using the Alkaline Isoelectric Point Method

Fifty grams of *B. fuscopurpurea* powder were weighed. Water was added at a mass-to-volume ratio of 1:25 and stirred to allow swelling for 4 h. The pH was adjusted to 9. Ultrasonic extraction was performed at 480 W for 20 min, then the extract was stirred at 50 °C for 3 h. The mixture was centrifuged at 5201× *g* for 15 min. The pH of the supernatant was adjusted to 4.2, then centrifuged at 5201× *g* for 15 min after standing at 4 °C for 12 h. The precipitate was freeze-dried to obtain red algae protein for subsequent use.

### 2.5. Preparation of Enzymatic Hydrolysates

Four enzymes were chosen for comparison, namely, papain, bromelain, neutral protease and alkaline protease. Red algae protein with pH 2.2 was used as the substrate. Optimal conditions of each enzyme were used to determine the most suitable hydrolytic enzyme through the determination of the antioxidant activity of the enzymatic hydrolysate. The preparation of the protein was performed at 20 mg/mL solution. An enzyme-to-substrate ratio ([E]/[S]) of 6846 IU/gprot was used in adding papain, bromelain, neutral protease, and alkaline protease. The digestion rate of the enzymes was performed at 50 °C in 2 h with a pH of 10 in alkaline protease and a pH of 7 in other enzymes. The antioxidant activity of the enzymatic hydrolysate was determined, and the enzyme producing the hydrolysate with the highest antioxidant capacity was selected as the hydrolytic enzyme.

*B. fuscopurpurea* protein was weighed to prepare a 20 mg/mL solution. Papain was added at an enzyme substrate ratio ([E]/[S]) of 6846 IU/gprot. The enzymatic digestion conditions were temperature 50 °C, pH 7.0, and digestion time of 2 h. After digestion, the enzyme was inactivated by heating at 100 °C for 5 min. The solution was concentrated under reduced pressure at 60 °C to 1/4 of the original volume. Ethanol was added to achieve a 70% ethanol concentration to remove enzymes, large-molecule peptones, and unhydrolyzed proteins. The mixture was centrifuged at 5201× *g* for 20 min and the supernatant was collected. The resulting ethanol solution of hydrolyzed *B. fuscopurpurea* peptides is obtained.

### 2.6. Fractionation by Ultrafiltration

Using ultrafiltration membranes with molecular weight cut-offs of 2 KDa and 10 KDa, peptide fractions below 2 KDa (BFPI) and between 2 KDa and 10 KDa (BFPII) were prepared. The two peptide fractions BFPI and BFPII were freeze-dried. The DPPH radical scavenging rate and the oxygen radical absorbance capacity (ORAC) value were determined.

### 2.7. Purification by Chromatography

The two peptide fractions, BFPI and BFPII, obtained above were separately passed through a 4 × 25 cm DEAE-52 anion exchange column. Elution was performed using a linear gradient of 0–2 M NaCl at a flow rate of 3.0 mL/min. The antioxidant activity of the resulting peptide fractions was measured. The peptide fraction with superior antioxidant activity was selected for further separation. Fractions below 2 kDa were eluted from a 2.5 × 60 cm Sephadex LH-20 column using deionized water at a flow rate of 3.0 mL/min. Peptide fractions between 2 kDa and 10 kDa were eluted from a 2.5 × 60 cm Sephadex G-75 column, also using deionized water at a flow rate of 3.0 mL/min.

### 2.8. Peptide Composition Analysis of Peptide Components

Peptides underwent reduction, alkylation, and desalting. The processed samples were analyzed using liquid chromatography–mass spectrometry (LC-MS/MS), yielding raw mass spectrometry data files. These files underwent de novo analysis with PEAKS Studio 10.6 to generate peptide sequence results. Raw files generated from LC-MS/MS data acquisition were opened in Xcalibur to visualize the total ion chromatograms. Peptides were selected for synthesis based on their abundance and confidence scores. The analysis system was equipped with a pre-column (300 μm i.d. × 5 mm, packed with Acclaim PepMap RPLC C18, 5 μm, 100 Å) and an analytical column (150 μm i.d. × 150 mm, packed with Acclaim PepMap RPLC C18, 1.9 μm, 100 Å). Mobile phase A consisted of 0.1% formic acid and 2% acetonitrile, and mobile phase B consisted of 0.1% formic acid and 80% acetonitrile. The analysis was performed at a flow rate of 600 nL/min, and the total analysis time per sample was 120 min.

### 2.9. DPPH, ABTS, and OH Radical Scavenging Assays

#### 2.9.1. DPPH Radical Scavenging Activity

The DPPH radical scavenging activity was determined according to the method of [15] with slight modifications. Briefly, 100 μL of the sample solution at various concentrations was mixed with 100 μL of a 0.2 mmol/L DPPH solution prepared in methanol. The mixture was incubated for 30 min in the dark at room temperature. The absorbance was then measured at 517 nm. Vitamin C or Trolox was used as a positive control. The scavenging activity was calculated using the following formula:

$$Scavenging\;Activity\left(\%\right)=(1-\frac{A_{sample}{-A}_{sample\;blank}}{A_{control}})\times 100$$

where A_sample_ is the absorbance of the sample with DPPH, A_sample blank_ is the absorbance of the sample with methanol (background), and A_control_ is the absorbance of DPPH with the sample solvent (control).

#### 2.9.2. ABTS Radical Scavenging Activity

The ABTS radical scavenging activity was measured using the method of [16]. First, the ABTS^+^ radical cation stock solution was produced by reacting equal volumes of 7 mmol/L ABTS solution and 2.45 mmol/L potassium persulfate (K_2_S_2_O_8_) solution in the dark at room temperature for 12–16 h. Prior to use, the stock solution was diluted with phosphate-buffered saline (PBS, pH 7.4) to an absorbance of 0.70 ± 0.02 at 734 nm (the ABTS working solution). For the assay, 20 μL of sample solution at various concentrations was mixed with 180 μL of the ABTS working solution. After 10 min of incubation in the dark at room temperature, the absorbance was measured at 734 nm. The calculation formula was the same as in Section 2.9.1.

#### 2.9.3. Hydroxyl Radical (•OH) Scavenging Activity

The hydroxyl radical scavenging activity was measured using the salicylic acid method based on the Fenton reaction [17]. The reaction mixture, in a total volume of 2.0 mL, contained 0.5 mL of sample solution, 0.5 mL of FeSO_4_ (9 mmol/L), 0.5 mL of salicylic acid–ethanol (9 mmol/L), and 0.5 mL of H_2_O_2_ (9 mmol/L). The mixture was incubated at 37 °C for 30 min. After the reaction, the absorbance was measured at 510 nm. The calculation was the same as in 2.9.1, where A_control_ was the absorbance of the reaction mixture without the sample, and A_sample blank_ was the absorbance of the sample mixture without H_2_O_2_.

### 2.10. ORAC Assay

The ORAC activity was measured based on the fluorescein method described by [18] in a 96-well black microplate. Briefly, 150 μL of fluorescein (working concentration 70 nmol/L) and 25 μL of the sample (or Trolox standard, or blank buffer) were added to the wells. The plate was pre-incubated at 37 °C for 10 min. The reaction was initiated by the rapid addition of 25 μL of AAPH solution dihydrochloride, with a working concentration of 12 mmol/L). The fluorescence decay was immediately monitored kinetically using a fluorescence microplate reader at 485 nm (excitation) and 528 nm (emission), with readings taken every 2 min for 90 min. Antioxidant activity was quantified by calculating the net area under the curve (Net AUC), and results were expressed as μmol of Trolox equivalents (TE) per gram of sample.

### 2.11. Cell Antioxidant Activity Assay

#### 2.11.1. CCK-8 Assay for Cell Viability

HepG2 cells were seeded at a density of 100 μL per well in DMEM medium (4 × 10^3^ cells per well) in a cell culture plate. The cells were incubated at 37 °C in a 5% CO_2_ incubator for 12 h. The CCK-8 assay was employed to evaluate the effects of H_2_O_2_ and peptides on HepG2 cell viability. HepG2 cells (4 × 10^3^ cells/well) were seeded into cell culture plates at a density of 100 μL/well. After 12 h of incubation, H_2_O_2_ was measured at final concentrations of 100, 200, 400, 800, 1200, and 1800 μmol/L, and 3.125, 6.25, 12.5, 25, 50, and 100 μmol/L. A blank control group was established by adding an equal volume of DMEM medium. The assays were performed after 48 h of incubation.

#### 2.11.2. Measurement of Oxidative Stress-Related Factors

The experiment was set up with the following five groups: a normal control group, model group, low-dose peptide intervention group, medium-dose peptide intervention group, and high-dose peptide intervention group. HepG2 cells (2 mL) were seeded at 1 × 10^5^ cells/well in a 6-well cell culture plate and cultured for 12 h at 37 °C in a 5% CO_2_ incubator. The normal control group and model group received 2 mL of the DMEM medium. The low-, medium-, and high-dose intervention groups received peptides at concentrations of 2, 8, and 30 μmol/L (3.2, 12.8, and 48 μg/mL), respectively, and were cultured for 24 h at 37 °C in a 5% CO_2_ incubator. The normal control group was added with 2 mL of the DMEM medium. The model group and intervention groups were each added with 800 μmol/L H_2_O_2_ (concentration determined by cell viability assay, using a concentration slightly below 50% survival rate, 800 μmol/L). After 4 h incubation, cells were washed twice with PBS and collected. Intracellular ROS levels, SOD (WST assay) activity, CAT (ammonium molybdate assay) activity, GSH-Px activity, and MDA (microplate assay) levels were determined according to the kit instructions (Nanjing Jiancheng Technology Co., Ltd.).

##### ROS (Chemical Fluorescence Method)

Reactive oxygen species (ROS) levels were detected using the DCFH-DA (2′,7′-dichlorofluorescin diacetate) fluorescent probe. DCFH-DA itself is non-fluorescent and can freely permeate the cell membrane. Once inside the cell, it is hydrolyzed by intracellular esterases to DCFH (dichlorofluorescin), which is membrane-impermeable and thus becomes trapped within the cells. In the presence of intracellular ROS, DCFH is oxidized to the highly fluorescent compound DCF (dichlorofluorescein). DCF exhibits maximal fluorescence at an excitation wavelength of 488 nm and an emission wavelength of 525 nm, and its fluorescence intensity is proportional to the intracellular ROS level.

##### SOD (WST Method) Superoxide Dismutase

WST-1 can react with the superoxide anion (O_2_^−^) generated by xanthine oxidase to produce a water-soluble formazan dye. Since SOD can catalyze the dismutation of superoxide anions, this reaction can be inhibited by SOD. Therefore, SOD activity is negatively correlated with the amount of formazan produced, and the enzyme activity can be calculated by colorimetric analysis of the WST-1 reaction product. The reaction product of WST-1 is a stable and water-soluble substance, which allows SOD activity to be measured by determining absorbance at a single time point. Moreover, it is not affected by certain common interfering factors, resulting in significantly improved detection performance compared with several other commonly used methods.

##### Catalase (CAT) Activity Assay (Ammonium Molybdate Method)

The decomposition reaction of H_2_O_2_ by catalase (CAT) can be rapidly terminated by adding ammonium molybdate. The remaining H_2_O_2_ then reacts with ammonium molybdate to form a pale yellow complex. The absorbance of this complex is measured at 405 nm, and the change in absorbance is used to calculate CAT activity.

##### Glutathione Peroxidase (GSH-Px)

Glutathione peroxidase (GSH-Px) catalyzes the reaction between hydrogen peroxide (H_2_O_2_) and reduced glutathione (GSH), producing water (H_2_O) and oxidized glutathione (GSSG). The activity of glutathione peroxidase is expressed by the rate of its enzymatic reaction. By measuring the consumption of reduced glutathione in this reaction, the enzyme’s activity can be determined. GSH-Px activity is expressed as the reaction rate of GSH catalysis. Since both substrates undergo redox reactions in the absence of enzyme (termed non-enzymatic reactions), the final calculation of enzyme activity must subtract the GSH reduction attributable to non-enzymatic reactions. For GSH quantification, GSH reacts with dithiothreitol to form the 5-thiodinitrobenzoate anion, which exhibits a stable yellow color. Measuring its absorbance at 412 nm allows for the calculation of the GSH concentration.

##### Cell MDA (Microplate Method) Malondialdehyde

Malondialdehyde (MDA), a degradation product of lipid peroxidation, can condense with thiobarbituric acid to form a red product exhibiting a maximum absorption peak at 532 nm.

#### 2.11.3. Nematode Synchronization Treatment

The M9 buffer, 1 M NaOH solution, and 4% NaClO solution were prepared using the sodium hypochlorite synchronization method. The insect-containing plates were washed with an M9 buffer, then the worm-containing wash solution was collected into sterile 1.5 mL centrifuge tubes. The tubes were centrifuged at 606× *g* for 1 min, the supernatant was discarded, and the pellet was resuspended in M9 buffer; this process was repeated until the liquid was clear. The supernatant was discarded. A total of 0.5 mL of NaOH solution, 1 mL of NaClO solution, and 3.5 mL of M9 buffer were mixed, and then added to the worm-containing tube. The tube was gently shaken until most worms were broken down or dissolved. The tube was centrifuged at 2426× *g* for 1 min and the supernatant was discarded. It was then washed with a M9 buffer, and this was repeated approximately 2–3 times until the chlorine odor disappeared. Finally, the eggs were transferred from the bottom of the tube to the edge of a clean Nematode Growth Medium (NGM) plate inoculated with OP50. The eggs were incubated overnight. The larvae were transferred to a new clean NGM plate inoculated with OP50 to complete synchronization.

#### 2.11.4. Acute Oxidative Stress in Nematodes

##### Nematode Lifespan Assay

Synchronized nematodes were randomly divided into 4 groups, each with 3 replicates and 30 individuals per plate. The groups included a control and peptide intervention group (2, 8, and 30 µmol/L). They were cultured at 20 °C in an incubator for 72 h. Nematodes were transferred from each group to fresh NGM containing 15 μL of 30% hydrogen peroxide (*v*/*v*) per 10 mL. Nematode survival counts were recorded hourly until all nematodes died. The average lifespan was calculated using the following formula based on survival and death times.

$$Average\;life\;expectancy=1/N\sum_{t=1}^{t}\left[\left(x_{t-1}-x_{t}\right)\times t\right]$$

In the equation, *x_t_* denotes the number of nematodes surviving at time *t*; *N* denotes the initial total number of nematodes.

##### Measurement of Oxidative-Stress-Related Factors in Nematodes

Synchronized nematodes were randomly divided into 5 groups, each with 3 replicates (approximately 2000 individuals per tube). These groups included a blank control, a model group, and groups treated with different peptide concentrations (2, 8, and 30 µmol/L). They were cultured in 2 mL of S-Complete medium containing OP50 at 20 °C with shaking at 1× *g* for 72 h. A total of 2 μL of 30% H_2_O_2_ were added and cultured at 20 °C for 24 h. They were centrifuged at 606× *g* for 1 min, the supernatant was discarded, and they were washed three times with 0.01 mol/L PBS. The sonicated nematodes were in 200 μL of PBS on ice, followed by centrifugation at 2426× *g*. The supernatant was collected and protein concentration, SOD, GSH-Px, and MDA were measured in the supernatant according to the kit instructions.

### 2.12. Statistical Analysis

All experimental data were analyzed using GraphPad Prism 8 software. The results are expressed as mean ± standard deviation (SD) from at least three independent experiments. Statistical comparisons between multiple groups were performed using a one-way analysis of variance (ANOVA) followed by Dunnett’s post hoc test to compare the treatment groups against the control. A *p*-value of less than 0.05 was considered statistically significant.

## 3. Results and Analysis

### 3.1. Extraction Yield and Purity

The freeze-dried precipitate yielded 3.47 g of crude *B. fuscopurpurea* protein, corresponding to an extraction yield of 6.94%. The protein content of the crude extract was 58.43 ± 0.36%, as determined by the Kjeldahl method.

### 3.2. Enzyme Selection

Following enzymatic hydrolysis, the hydrolysate was diluted fivefold to determine DPPH radical scavenging activity and ORAC values. As shown in Table 1, the hydrolysate prepared using papain exhibited the highest antioxidant activity. Therefore, papain was selected as the enzyme for preparing antioxidant peptides.

### 3.3. Ultrafiltration Separation

Ultrafiltration membranes with molecular weight cut-offs of 2 KDa and 10 KDa were used to prepare peptide fractions below 2 KDa and between 2 KDa and 10 KDa. Freeze-drying yielded two peptide fractions: BFPI (below 2 KDa) and BFPII (2 KDa–10 KDa). DPPH radical scavenging activity and ORAC values were measured. As shown in Table 1, the two ultrafiltration-prepared peptide fractions exhibited no significant differences in DPPH radical scavenging activity or ORAC values. Therefore, further separation of the BFPI and BFPII fractions was conducted.

### 3.4. Chromatographic Separation

**Figure 1:**
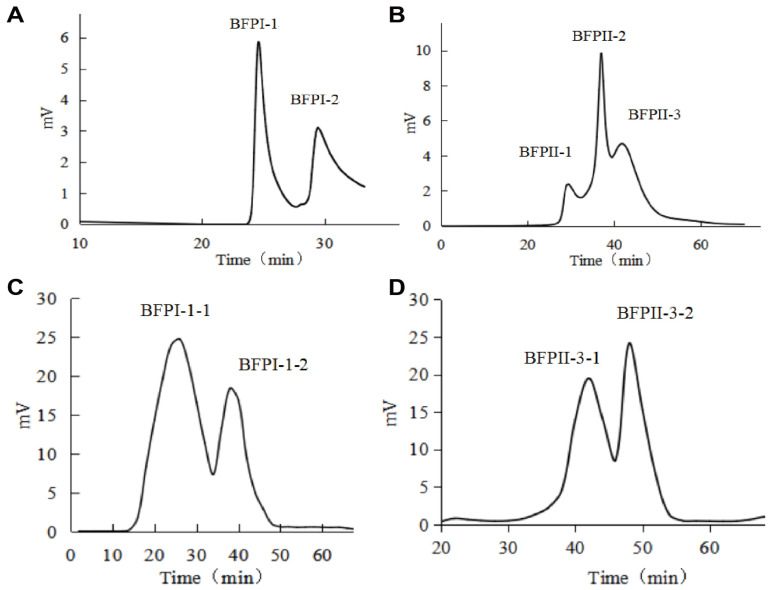
Chromatographic purification profiles of peptide fractions. (**A**) Anion exchange chromatography profile of BFPI on a DEAE-52 column. (**B**) Anion exchange chromatography profile of BFPII on a DEAE-52 column. (**C**) Gel filtration chromatography profile of the BFPI-1 fraction on a Sephadex LH-20 column. (**D**) Gel filtration chromatography profile of the BFPII-3 fraction on a Sephadex G-75 column. Conditions: for (**A**,**B**), a DEAE-52 column (4 × 25 cm) was eluted with a linear gradient of 0–2 mol/L NaCl at 3.0 mL/min. For (**C**), a Sephadex LH-20 column (2.5 × 60 cm) and for (**D**), a Sephadex G-75 column (2.5 × 60 cm) were used; both were eluted with deionized water at 3.0 mL/min.

The two polypeptide fractions, BFPI and BFPII, obtained above were passed through a 4 × 25 cm DEAE-52 anion exchange column. Elution was performed using a linear gradient of 0–2 mol/L NaCl at a flow rate of 3.0 mL/min. BFPI yielded two fractions: BFPI-1 and BFPI-2, while BFPII yielded three components: BFPII-1, BFPII-2, and BFPII-3 (Figure 1A,B). Each component was desalted by dialysis and freeze-dried. The DPPH radical scavenging rates and ORAC values for each component are shown in Table 1.

The BFPI-1 and BFPII-3 peptide fractions, which exhibited superior DPPH radical scavenging and ORAC values, were selected for subsequent separation. The BFPI-1 fraction was run through a 2.5 × 60 cm Sephadex LH-20 column, and eluted with deionized water at a flow rate of 3.0 mL/min. The BFPII-3 fraction was run through a 2.5 × 60 cm Sephadex G-75 column, eluted with deionized water at a flow rate of 3.0 mL/min. The BFPI-1 yielded two fractions: BFPI-1-1 and BFPI-1-2; the BFPII-3 yielded two fractions, BFPII-3-1 and BFPII-3-2 (Figure 1C,D). After dialysis desalting and freeze-drying, the DPPH radical scavenging activity and ORAC values were determined (Table 1). The fractions BFPI-1-1 and BFPII-3-2 exhibited excellent DPPH radical scavenging and ORAC values.

### 3.5. Peptide Composition Analysis and Activity Assay of Synthetic Peptide Fragments

**Figure 2:**
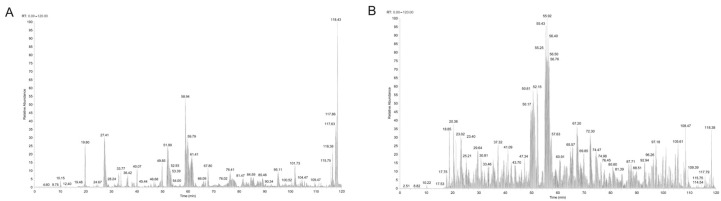
LC-MS/MS identification of peptide fractions and in vitro antioxidant activities of synthetic peptides. (**A**) Total ion chromatogram (TIC) of the BFPI-1-1 fraction. (**B**) Total ion chromatogram (TIC) of the BFPII-3-1 fraction.

The peptide compositions of BFPI-1-1 and BFPII-3-1—the peptide components exhibiting the strongest antioxidant effects—were determined using liquid chromatography–mass spectrometry (LC-MS/MS). The total ion chromatogram indicates that both the BFPI-1-1 and BFPII-3-1 fractions are mixtures without a dominant peptide of significantly higher abundance (Figure 2A,B). Molecular weight analysis reveals a broad distribution of peptide molecular weights in the samples, with the range of higher-abundance peptides spanning 300–2000 Da and containing 3 to 20 amino acids. Therefore, de novo peptide sequencing was performed without enzymatic digestion. This sequencing utilized Thermo’s Orbitrap Fusion Lumos mass spectrometer. By manually de-scrambling the secondary fragmentation patterns of the peptides, the amino acid sequences were deduced. For the BFPI-1-1 fraction, the top 4 abundant peptides—FLPDLT, VLPYAK, FGEPAF, and KFLTN—were selected for synthesis. For the BFPII-3-1 fraction, the top six peptides by abundance—YPCW, TTFTT, EEAP, NKLSSY, GYPYK, and EAAK—were selected for synthesis. The sample concentration for the DPPH and ABTS radical scavenging assays was 0.2000 mg/mL, while the concentration for the OH radical scavenging assay was 1.000 mg/mL (Table 2).

The four peptides VLPYAK, YPCW, NKLSSY, and GYPYK, which demonstrated excellent ABTS radical scavenging activity in Table 2 above, were selected. Their IC_50_ values for DPPH and ABTS radical scavenging and ORAC values, were determined (Table 3). The peptides YPCW and GYPYK exhibited the most outstanding performance and were selected for further experiments in cells and nematodes.

### 3.6. Cellular Antioxidant Activity Assay

#### 3.6.1. Effects of H_2_O_2_ and Peptides on Cell Survival Rate

**Figure 3:**
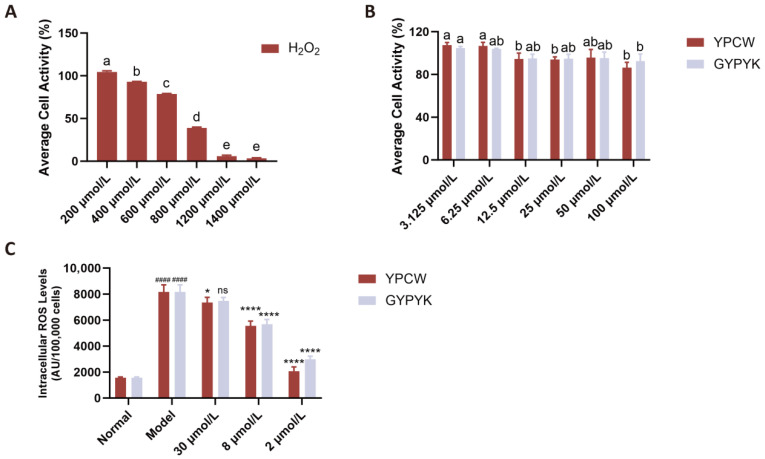
Effects of YPCW and GYPYK peptides on HepG2 cell viability and intracellular ROS levels induced by H_2_O_2_. (**A**) Effects of different concentrations of H_2_O_2_ on the viability of HepG2 cells (oxidative stress model establishment). (**B**) Effects of different concentrations of YPCW and GYPYK peptides on the viability of HepG2 cells (cytotoxicity assay). (**C**) Inhibitory effects of YPCW and GYPYK peptides on intracellular reactive oxygen species (ROS) levels in H_2_O_2_-induced (Model) HepG2 cells. All data are presented as the mean ± standard deviation (SD). In (**A**,**B**), bars with different lowercase letters indicate significant differences between groups (*p* < 0.05). In (**C**), ^####^ *p* < 0.0001 vs. the normal control group; * *p* < 0.05, **** *p* < 0.0001 vs. the Model group; ns indicates no significant difference.

The CCK-8 assay was used to assess the impact of H_2_O_2_ on HepG2 cell viability, with 48 h culture results shown in Figure 3A. Cells treated with 800 μmol/L H_2_O_2_ exhibited a viability rate of 38.84 ± 0.87%, below the 50% threshold, confirming significant cellular damage. This concentration was selected to establish the damage model. The effect of the peptide YPCW on HepG2 cell survival is shown in Figure 3B. The levels of the peptide YPCW used, such as 3.125 to 100 μmol/L, did not affect the cell survival rate significantly; hence, this can be used in further experiments.

#### 3.6.2. Effects of Peptides on ROS Levels in HepG2 Cells

ROSs, superoxide radicals, hydrogen peroxide, and their subsequent products, including peroxides and hydroxyl radicals, are involved in cellular growth, proliferation, development, differentiation, aging, apoptosis, and many other physiological and pathological events. One of the strategies of fighting oxidative stress is to eliminate ROS in the body. In this experiment, the level of ROS in the model group increased significantly to 5.19 times that of the normal control group, which proves that there was massive production of intracellular ROS in the presence of H_2_O_2_. YPCW or GYPYK used as pre-treatment had a profound effect on reducing ROS. The relationship between dose and response was determined by the relationship between the two concentrations of 2–30 μmol/L, as illustrated in Figure 3C, where YPCW showed a slightly better performance than GYPYK.

#### 3.6.3. Effects of Peptides on Oxidative-Stress-Related Factors in HepG2 Cells

Antioxidant enzymes help to minimize the concentration of oxygen free radicals in the body and guard cells against oxidative damage [19], and these enzymes include SOD, GSH-Px, and CAT. The experiment was formulated using five groups namely normal control group, model group, low-dose peptide intervention group, medium-dose peptide intervention group, and high-dose peptide intervention group. The HepG2 cells (2 mL at 1 × 10^5^ cells/well) were incubated in 6-well cell culture plates and allowed to grow after 12 h at 37 °C in a 5% CO_2_ incubator. DMEM medium of 2 mL was added to the normal control group and model group. The intervention groups (low-, medium-, and high-dose) were exposed to peptides of 2, 8, and 30 μmol/L, incubated in a 5% CO_2_ incubator at 37 °C, and incubated over a period of 24 h. DMEM medium was added to the normal control group (2 mL). H_2_O_2_ 800 μmol/L was added to the model group and the intervention groups in each of the wells. An incubation period of 4 h was followed by a two-rinsed collection of cells with PBS. The SOD, CAT, and GSH-Px enzyme activity and MDA levels in intracellular were measured based on the instructions in the kit.

**Figure 4:**
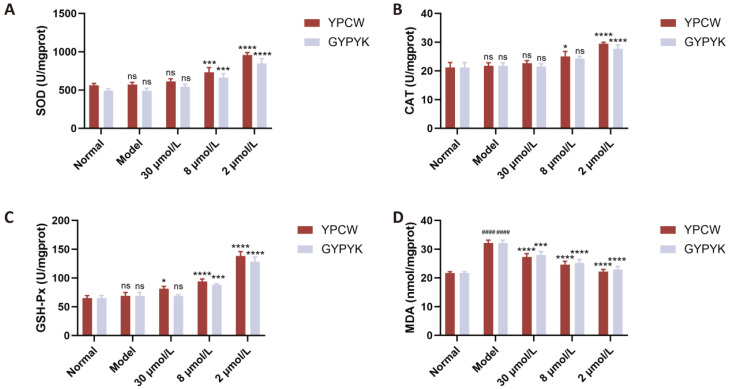
Effects of YPCW and GYPYK peptides on oxidative-stress-related factors in HepG2 cells. (**A**) Activity of superoxide dismutase (SOD). (**B**) Activity of catalase (CAT). (**C**) Activity of glutathione peroxidase (GSH-Px). (**D**) Content of malondialdehyde (MDA). All data are presented as the mean ± standard deviation (SD). ^####^ *p* < 0.0001 vs. the normal control group; * *p* < 0.05, *** *p* < 0.001, **** *p* < 0.0001 vs. the model group; ns indicates no significant difference.

Cellular oxidative damage can also be alleviated by antioxidants that control the levels of antioxidant enzymes and antioxidant-related genes in the body. As a number of studies have shown, the intracellular antioxidant enzyme system is a vital protection in the face of oxidative stress areas. It works by transforming highly reactive O_2_^−^ and H_2_O_2_^+^ into less reactive O_2_ and H_2_O, respectively, via the action of antioxidant enzymes, including SOD, CAT, and GSH-Px. Thus, the changes in the activity of intracellular antioxidant enzymes are used as indicators of the antioxidant activity of a substance [20,21]. The model and normal control groups did not show any significant differences in the SOD, CAT, or GSH-Px activity (Figure 4), thus showing that the oxidative stress, which is generated through the cellular damage, increased the activity or expression of these enzymes, and, therefore, retained the residual activity at the same level as the normal control group. SOD, CAT and GSH-Px activities were greatly enhanced dose dependently following peptide intervention. The Keap1/Nrf2 pathway was also triggered by the antioxidant peptides. In the absence of a disease state, the Kelch-like epoxychloropropane-1-protein Kelch1, binds to nuclear factor erythroid 2-related factor 2 (Nrf2) extranucleally. At the time of ROS attack, Keap1/Nrf2 dissociates, releasing Nrf2 into the nucleus that is bound to the antioxidant-responsive element (ARE) thus triggering the expression of phase II detoxification enzymes and antioxidants (SOD, GSH-Px, CAT) [22,23]. The MDA content in cells was greater in the model group than the normal control group showing that oxidative damage raised the level of MDA in the products of lipid peroxidation degradation. The significant reduction in MDA levels was caused by varying intervention with peptides YPCW and GYPYK.

#### 3.6.4. Assessment of Peptide Effects on Nematode Lifespan

Synchronized nematodes were randomly divided into four groups, each with three replicates and 30 individuals per plate. The treatments were an injury control group, a peptide intervention group (4, 16, and 60 µmol/L). The nematodes were then transferred to new NGM, with 15 μL/10 mL 30% (*v*/*v*) H_2_O_2_ after 72 h of growing in an artificial climate chamber at 20 °C. The number of survivors was taken at every hour up to ultimate death. YPCW and GYPYK had a significant positive effect on the lifespan of oxidative stress-damaged nematodes (Table 4), and a dose–response correlation was observed at the concentrations of 2–30 μmol/L.

#### 3.6.5. Effects of Peptides on Oxidative-Stress-Related Factors in Nematodes

Synchronized nematodes were randomly divided into five groups with three replicates per group, each tube containing approximately 2000 individuals. Groups included a blank control, a model group, and intervention groups treated with peptides at different concentrations (2, 8, and 30 µmol/L). They were cultured in 2 mL of S-Complete medium containing OP50 at 20 °C with shaking at 1× *g* for 72 h. A total of 2 μL of 30% H_2_O_2_ were added, cultured at 20 °C for 24 h, then centrifuged at 606× *g* for 1 min. The supernatant was discarded, washed three times with 0.01 mol/L PBS, and then 200 μL PBS was added. The mixture was sonicated in ice water and centrifuged at 2426× *g*, and the supernatant was collected. Protein concentration, SOD, CAT, GSH-Px enzyme activity, and MDA concentration in the supernatant were measured according to the kit instructions.

**Figure 5:**
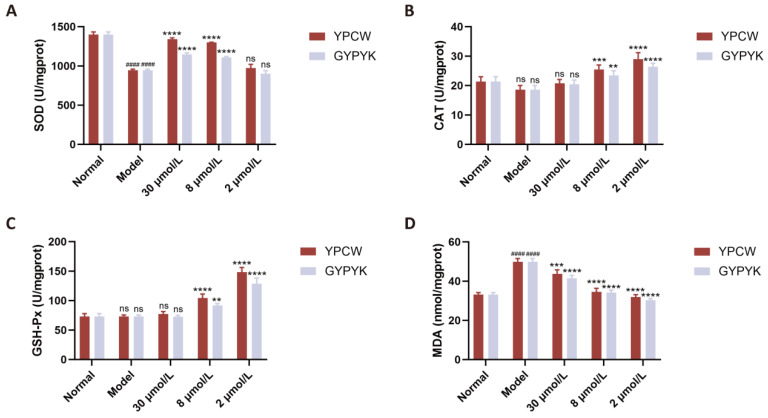
Effects of YPCW and GYPYK peptides on oxidative-stress-related factors in nematodes. (**A**) Activity of superoxide dismutase (SOD). (**B**) Activity of catalase (CAT). (**C**) Activity of glutathione peroxidase (GSH-Px). (**D**) Content of malondialdehyde (MDA). All data are presented as the mean ± standard deviation (SD). ^####^ *p* < 0.0001 vs. the normal control group; ** *p* < 0.01, *** *p* < 0.001, **** *p* < 0.0001 vs. the model group; ns indicates no significant difference.

As shown in Figure 5, significant differences (*p* < 0.05) were observed in SOD and CAT activities between the normal control and model groups of nematodes, while no significant difference (*p* > 0.05) was found in GSH-Px activity. Following YPCW and GYPYK intervention, SOD, CAT, and GSH-Px enzyme activities significantly increased, with a pronounced dose–response effect consistent with the cellular experiment results in Section 3.6.3. MDA concentrations in nematodes were significantly higher in the model group than in the normal control group, indicating that oxidative damage increased MDA levels among lipid peroxidation degradation products. Intervention with peptides YPCW and GYPYK significantly reduced MDA levels, further validating the cellular experiment results in Section 3.5.

## 4. Results and Discussion

In this study, two novel antioxidant peptides, YPCW and GYPYK, were successfully isolated from *B. fuscopurpurea* papain hydrolysate. The in vitro assays demonstrated significant antioxidant potential. Notably, YPCW exhibited a strong ABTS radical scavenging IC50 of 2.52 µg/mL, and GYPYK showed a high ORAC value of 5003 µmol TE/g. These results highlight the potential of *B. fuscopurpurea* as a high-quality protein source for generating bioactive peptides.

While the absolute potency of these peptides can be challenging to assess without a uniform positive control in all assays, the in vitro activity values obtained are significant. Furthermore, the strong bioactivity is supported by their amino acid composition. The presence of tyrosine (Y) in both peptides is well-established as a key contributor to radical scavenging due to its phenolic group acting as a hydrogen donor. This observation is in agreement with the structure–activity relationship reviews by Zhang et al. [11] and Qoms et al. [8], which confirmed that peptides containing aromatic amino acids (Tyr, Trp, Phe) generally exhibit superior radical scavenging abilities due to their electron-donating properties. The peptide YPCW is particularly notable as it also contains cysteine (C), which provides a highly reactive sulfhydryl group (similar to glutathione), and tryptophan (W), whose indole ring is also a known potent scavenger. The presence of proline (P) in both peptides likely enhances their stability and radical scavenging capacity.

More significantly, this study demonstrated that the peptides provide robust protection in cellulo and in vivo. In both H_2_O_2_-stressed HepG2 cells and *C. elegans*, YPCW and GYPYK not only reduced intracellular ROS and MDA levels, but also enhanced the activity of the endogenous antioxidant enzymes SOD, CAT, and GSH-Px. This suggests that their primary protective mechanism in vivo may be the upregulation of the host’s innate cellular defense system, rather than just direct radical scavenging. Based on these limitations and findings, future work should focus on elucidating the specific molecular pathways involved in this protective effect, specifically by verifying the translocation of Nrf2 and the expression of downstream ARE-mediated genes. Additionally, studies on the gastrointestinal stability and bioavailability of YPCW and GYPYK are necessary to assess their practical application.

In conclusion, this study successfully identified YPCW and GYPYK from *B. fusco-purpurea* as potent antioxidant agents. They operate via a dual mechanism with direct radical scavenging and enhancing endogenous enzymatic defenses. These findings validate their potential as novel ingredients for functional foods or nutraceuticals to combat oxidative stress. Future work should focus on elucidating the specific molecular pathways involved in this protective effect.

## Figures and Tables

**Table 1:** DPPH radical scavenging rates and ORAC values of different enzymatic products and purified components at various stages.

Sample	Concentration (mg/mL)	DPPH Radical Scavenging Rate (%)	ORAC Value (μmol Trolox/g)
**Enzymatic hydrolysate**			
Papain	/	40.25 ± 2.23	386 ± 12
Bromelain	/	19.47 ± 1.04	204 ± 14
Neutral protease	/	8.85 ± 1.11	89 ± 8
Alkaline protease	/	13.55 ± 2.06	158 ± 12
**Primary components**			
BFPI	1.0	55.69 ± 0.96	516 ± 52
BFPII	1.0	59.14 ± 1.23	651 ± 36
**Secondary sub-components**			
BFPI-1	0.5	51.02 ± 1.76	1054 ± 87
BFPI-2	0.5	12.10 ± 0.24	451 ± 55
BFPII-1	0.5	10.87 ± 0.21	402 ± 36
BFPII-2	0.5	32.85 ± 1.36	856 ± 88
BFPII-3	0.5	61.54 ± 1.98	1265 ± 95
**Tertiary sub-components**			
BFPI-1-1	0.2	40.32 ± 0.35	1879 ± 120
BFPI-1-2	0.2	8.78 ± 0.08	558 ± 66
BFPII-3-1	0.2	50.54 ± 1.12	2014 ± 154
BFPII-3-2	0.2	15.47 ± 0.98	966 ± 69

**Table 2:** Free radical scavenging activity of the synthesized 10 peptides against DPPH, ABTS, and OH radicals.

	DPPH Radical Scavenging Rate (%)	ABTS Radical Scavenging Rate (%)	OH Radical Scavenging Rate (%)
FLPDLT	0	6.68 ± 1.10	0
VLPYAK	0	85.33 ± 0.76	19.74 ± 4.47
FGEPAF	0	8.54 ± 1.56	4.81 ± 5.00
KFLTN	0	2.86 ± 2.16	0
YPCW	29.73 ± 0.88	99.22 ± 0.42	3.91 ± 2.43
TTFTT	0	1.13 ± 1.31	17.29 ± 2.93
EEAP	0	1.13 ± 1.98	0
NKLSSY	0	94.18 ± 0.04	2.65 ± 5.75
GYPYK	0	95.28 ± 0.15	28.38 ± 5.48
EAAK	0	1.71 ± 1.91	11.58 ± 2.17

**Table 3:** IC_50_ values and ORAC values for the scavenging of DPPH and ABTS radicals by the four synthesized peptides.

	DPPH Radical Scavenging IC_50_ Value (µg/mL)	ABTS Radical Scavenging IC_50_ Value (µg/mL)	ORAC Value (μmol TE/g)	ORAC Value (μmol Trolox/μmol)	Molecular Weight
VLPYAK	/	7.02 ± 0. 87	1929 ± 147	1.35 ± 0.10	690.4182
YPCW	20.95 ± 0.33	2.52 ± 0.37	5187 ± 78	3.24 ± 0.05	625.2562
NKLSSY	/	5.40 ± 0.62	1797 ± 30	1.28 ± 0.02	712.3862
GYPYK	/	4.13 ± 0.67	5003 ± 190	3.35 ± 0.13	669.3251

**Table 4:** Effects of YPCW and GYPYK on the lifespan of nematodes under oxidative stress damage.

		Damage Control Group	2 μmol/L	8 μmol/L	30 μmol/L
YPCW	Average lifespan/h	5.24 ± 0.05	5.47 ± 0.03	6.20 ± 0.10	6.63 ± 0.13
	Lifespan extension rate/%	/	4.33 ± 0.64	18.32 ± 1.91	26.59 ± 2.54
GYPYK	Average lifespan/h	5.24 ± 0.05	5.30 ± 0.03	5.92 ± 0.02	6.16 ± 0.07
	Lifespan extension rate/%	/	1.15 ± 0.64	13.02 ± 0.37	17.47 ± 1.32

## Data Availability

The original contributions presented in this study are included in the article. Further inquiries can be directed to the corresponding author.

## References

[1] Li Y.L., Wang J., Pu J.Q., Hu C.M., Zhou W., Brodie J., Yang L.E. (2025). History and advances in the taxonomy of the Bangiaceae (Rhodophyta) and new evidence to support splitting the genus *Pyropia*. Eur. J. Phycol..

[2] Niu C., Wang W., Yao H., Liang Z., Zhang P., Lu X. (2023). Ascorbate− glutathione cycle involving in response of *Bangia fuscopurpurea* (Bangiales, Rhodophyta) to hyposalinity. Front. Mar. Sci..

[3] Singh S., Nirban R., Dutta T. (2021). MTS1338 in *Mycobacterium tuberculosis* promotes detoxification of reactive oxygen species under oxidative stress. Tuberculosis.

[4] Sharma P., Nandave M., Nandave D., Yadav S., Vargas-De-La-Cruz C., Singh S., Tandon R., Ramniwas S., Behl T. (2023). Reactive oxygen species (ROS)-mediated oxidative stress in chronic liver diseases and its mitigation by medicinal plants. Am. J. Transl. Res..

[5] Jîtcă G., Ősz B.E., Tero-Vescan A., Miklos A.P., Rusz C.M., Bătrînu M.G., Vari C.E. (2022). Positive aspects of oxidative stress at different levels of the human body: A review. Antioxidants.

[6] Leyane T.S., Jere S.W., Houreld N.N. (2022). Oxidative stress in ageing and chronic degenerative pathologies: Molecular mechanisms involved in counteracting oxidative stress and chronic inflammation. Int. J. Mol. Sci..

[7] Kıran T.R., Otlu O., Karabulut A.B. (2023). Oxidative stress and antioxidants in health and disease. J. Lab. Med..

[8] Qoms M.S., Wong S.K., Mohd Fauzi N., Husain K., Makpol S., Tan J.K. (2025). Microalgae-derived peptides targeting lifestyle-related diseases: Discovery, mechanisms, structure–activity relationships, and structural modifications. Antioxidants.

[9] Tang Y., Nie T., Zhang L., Liu X., Deng H. (2025). Peptides in cosmetics: From pharmaceutical breakthroughs to skincare innovations. Cosmetics.

[10] Zaky A.A., Simal-Gandara J., Eun J.B., Shim J.H., Abd El-Aty A.M. (2022). Bioactivities, applications, safety, and health benefits of bioactive peptides from food and by-products: A review. Front. Nutr..

[11] Zhang Y., Li Y., Quan Z., Xiao P., Duan J.A. (2024). New insights into antioxidant peptides: An overview of efficient screening, evaluation models, molecular mechanisms, and applications. Antioxidants.

[12] Andrés C.M.C., Pérez de la Lastra J.M., Munguira E.B., Juan C.A., Pérez-Lebeña E. (2025). The multifaceted health benefits of broccoli—A review of glucosinolates, phenolics and antimicrobial peptides. Molecules.

[13] Lu Z., Sun N., Dong L., Gao Y., Lin S. (2022). Production of bioactive peptides from sea cucumber and its potential health benefits: A comprehensive review. J. Agric. Food Chem..

[14] Jia L., Wang L., Liu C., Liang Y., Lin Q. (2021). Bioactive peptides from foods: Production, function, and application. Food Funct..

[15] Brand-Williams W., Cuvelier M.E., Berset C. (1995). Use of a free radical method to evaluate antioxidant activity. LWT-Food Sci. Technol..

[16] Re R., Pellegrini N., Proteggente A., Pannala A., Yang M., Rice-Evans C. (1999). Antioxidant activity applying an improved ABTS radical cation decolorization assay. Free Radic. Biol. Med..

[17] Smirnoff N., Cumbes C.N. (1989). Hydroxyl radical scavenging activity of compatible solutes. Phytochemistry.

[18] Huang D., Ou B., Hampsch-Woodill M., Flanagan J.A., Prior R.L. (2002). High-throughput assay of oxygen radical absorbance capacity (ORAC) using a multichannel liquid handling system coupled with a microplate fluorescence reader in 96-well format. J. Agric. Food Chem..

[19] Cai X., Chen S., Liang J., Tang M., Wang S. (2021). Protective effects of crimson snapper scales peptides against oxidative stress on *Drosophila melanogaster* and the action mechanism. Food Chem. Toxicol..

[20] Zhang M., Zhang H., Li H., Lai F., Li X., Tang Y., Min T., Wu H. (2016). Antioxidant mechanism of betaine without free radical scavenging ability. J. Agric. Food Chem..

[21] Liao W., Ning Z., Chen L., Wei Q., Yuan E., Yang J., Ren J. (2014). Intracellular antioxidant detoxifying effects of diosmetin on 2, 2-azobis (2-amidinopropane) dihydrochloride (AAPH)-induced oxidative stress through inhibition of reactive oxygen species generation. J. Agric. Food Chem..

[22] Wu D., Li M., Ding J., Zheng J., Zhu B., Lin S. (2020). Structure-activity relationship and pathway of antioxidant shrimp peptides in a PC12 cell model. J. Funct. Foods.

[23] Hou D.X., Korenori Y., Tanigawa S., Yamada-Kato T., Nagai M., He X., He J. (2011). Dynamics of Nrf2 and Keap1 in ARE-mediated NQO1 expression by wasabi 6-(methylsulfinyl) hexyl isothiocyanate. J. Agric. Food Chem..

